# Reconstruction of Genome-Scale Active Metabolic Networks for 69 Human Cell Types and 16 Cancer Types Using INIT

**DOI:** 10.1371/journal.pcbi.1002518

**Published:** 2012-05-17

**Authors:** Rasmus Agren, Sergio Bordel, Adil Mardinoglu, Natapol Pornputtapong, Intawat Nookaew, Jens Nielsen

**Affiliations:** Department of Chemical and Biological Engineering, Chalmers University of Technology, Gothenburg, Sweden; The Pennsylvania State University, United States of America

## Abstract

Development of high throughput analytical methods has given physicians the potential access to extensive and patient-specific data sets, such as gene sequences, gene expression profiles or metabolite footprints. This opens for a new approach in health care, which is both personalized and based on system-level analysis. Genome-scale metabolic networks provide a mechanistic description of the relationships between different genes, which is valuable for the analysis and interpretation of large experimental data-sets. Here we describe the generation of genome-scale active metabolic networks for 69 different cell types and 16 cancer types using the INIT (Integrative Network Inference for Tissues) algorithm. The INIT algorithm uses cell type specific information about protein abundances contained in the Human Proteome Atlas as the main source of evidence. The generated models constitute the first step towards establishing a Human Metabolic Atlas, which will be a comprehensive description (accessible online) of the metabolism of different human cell types, and will allow for tissue-level and organism-level simulations in order to achieve a better understanding of complex diseases. A comparative analysis between the active metabolic networks of cancer types and healthy cell types allowed for identification of cancer-specific metabolic features that constitute generic potential drug targets for cancer treatment.

## Author Summary

Many serious diseases have a strong metabolic component. The abnormal metabolic states of diseased cells could therefore be targets for treatment. However, metabolism is a highly complex and interconnected system in which thousands of metabolic reactions occur simultaneously in any given cell type. In order to understand how metabolism of a diseased cell differs from its healthy counterpart we must therefore study the system as a whole. We have developed an algorithm that integrates several types of data in order to generate active metabolic networks; catalogues of the metabolic reactions that are likely to be active in a given cell type. We applied this algorithm to data for 69 healthy cell types and 16 cancer cell types. These metabolic networks can form the basis for simulation of metabolic interactions between organs or as scaffolds for interpretation of high-throughput data. We used these networks to perform an analysis between cancer and healthy cell types in order to identify cancer specific metabolic features that constitute potential drug targets. Several of the resulting targets were already known and used clinically, but we also found high-ranking reactions and metabolites which have not yet been investigated as drug targets.

## Introduction

Abnormal metabolic states are at the origin of many diseases such as diabetes, hypertension, hearth diseases and cancer, which can be seen in many aspects as a metabolic disease. Cancer and coronary diseases are the two main causes of death in the developed countries. It is expected that by 2030 close to 200 million persons (33% of the total population) will be obese in the EU alone, and many of these will have one or more of the following co-morbidities: diabetes, hypertension, heart disease and increased risk of cancer, and the direct (medical treatment) and indirect (inability to work) costs are estimated to amount to more than €100 billion per year [1], [2]. The molecular mechanisms involved in these kinds of diseases are complex and in many cases different underlying molecular causes lead to the same disease phenotypes. A good understanding of human metabolism in different human cell types, whole tissues, and the interactions between them is therefore a necessary step towards efficient diagnosis and treatment of these diseases. Metabolism is, however, complex and involves a very large number of individual reactions that are highly interconnected through the sharing of common metabolites [3]. Understanding the function of metabolism therefore requires analysis of the complete metabolic network, and this is best done through the use of so-called genome-scale metabolic models (GEMs) [4], [5],[6].

There are three generic genome-scale human metabolic networks currently available, namely Recon1 [7], the Edinburgh Human Metabolic Network (EHMN) [8] and HumanCyc [9]. These reconstructions, however, are not tissue specific, which prevents their applicability to the study of particular human cell types or diseases. Tissue specific transcription profiles were used to generate tissue specific models for 10 different human tissues [10], which are subsets of Recon1, but these networks were not sufficiently flexible to explore the metabolic states of the tissues under various genetic and physiological conditions [11]. The same group later proposed a different algorithm that combines transcriptomic and proteomic data to generate a more flexible liver specific metabolic model [11], also using Recon1 as a template. Besides the mentioned automatically generated models, an extensive effort led to the publication of a manually reconstructed and annotated liver specific metabolic model referred as HepatoNet1 [12]. Models have also been developed for kidney [13], brain [14], erythrocytes [15] and alveolar macrophages [16]. Computational methods used to construct cell type specific metabolic models aim to integrate the evidence about the presence or absence of metabolic enzymes in a particular cell type, while at the same time maintaining a well-connected network (e.g. metabolites consumed in one reaction should be able to be produced in another reaction or to be taken up from the cell environment). Transcriptome data are often noisy and differences in mRNA expression are not absolute but relative to a reference condition, and in most cases do not correlate well with enzyme levels [17]. In the frame of the Human Protein Atlas (HPA) [18], [19], [20] cell type specific high quality proteomic data are being generated based on specific antibodies, and this represents an essential source for protein evidence in different human cell types.

**Figure 1:**
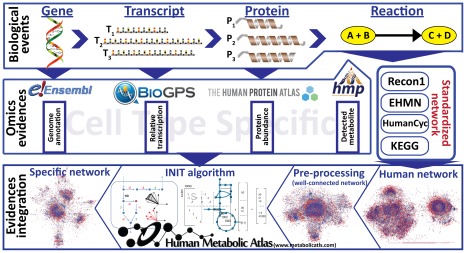
General pipeline used in the reconstruction of cell specific genome-scale metabolic networks. Biological information at the genome, transcriptome, proteome and metabolome levels contained in publicly available databases and generic human GEMs (Recon1, EHMN, HumanCyc) is integrated to form a generic human metabolic network, which is processed in order to obtain the connected *iHuman1512* network. Subsequently, the cell type specific evidence is used to generate cell type specific subnetworks using the INIT algorithm.

**Figure 2:**
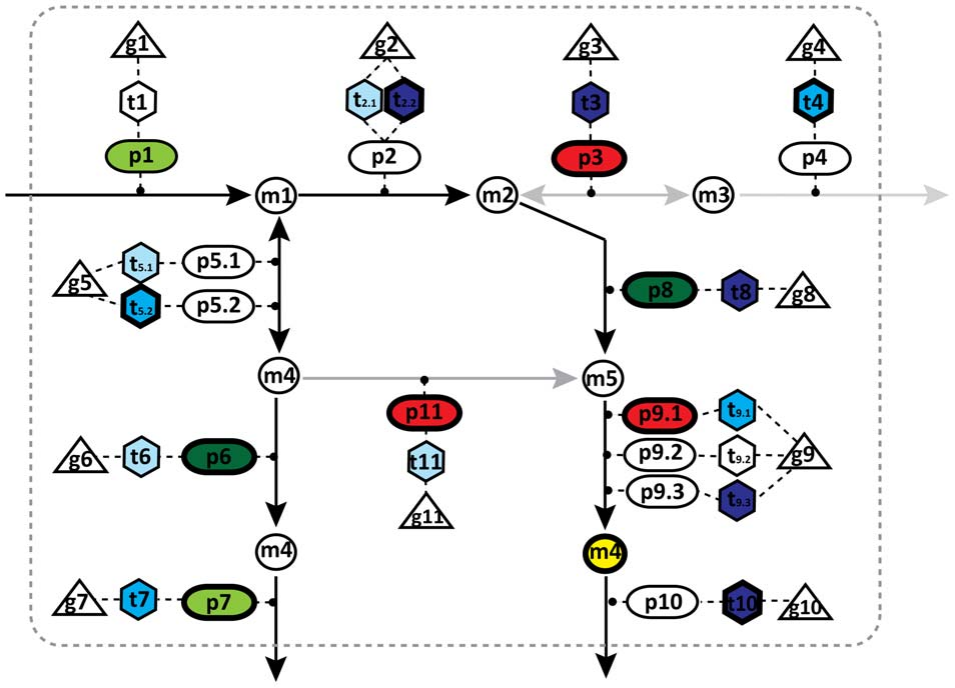
Illustration of the principles of the INIT algorithm. The hierarchical structure of GEMs is characterized by its gene-transcript-protein-reaction (GTPR) associations. In GEMs, each metabolic reaction is associated to one or more enzymes, which in turn are associated to transcripts and genes. Depending on the evidence for presence/absence of a given enzyme/gene in a cell type, a score can be calculated for the reaction(s) catalyzed by that enzyme. The HPA evidence scores are illustrated as red, light, medium and dark green representing negative, weak, moderate and strong evidence, respectively. The transcriptome evidence scores (GeneX), which are illustrated as red, light, medium, and dark blue representing low, medium and high expression, respectively. No evidence is present as white object. For some metabolites (yellow filled circle), metabolomic data are available to prove that they are present in the considered cell type. The aim of the algorithm is to find a sub-network in which the involved genes/proteins have strong evidence supporting their presence in the cell type under consideration. This is done by maximizing the sum of evidence scores. All the included reactions should be able to carry a flux and all the metabolites observed experimentally should be synthesized from precursors that the cell is known to take up. The bold lines represent the resulting network after optimization.

Here we present a pipeline for automatic identification of expressed cell type specific genome-scale metabolic networks (Figure 1). A key element of the pipeline is the INIT (Integrative Network Inference for Tissues) algorithm (Figure 2), which relies on the HPA as the main evidence source for assessing the presence or absence of metabolic enzymes in each of the human cell types that are present in the HPA. Tissue specific gene expression [21] was used as an extra source of evidence in INIT. Metabolomic data from the Human Metabolome Database (HMDB) [22] are also used as constraints in such a way that if a metabolite has been found in a particular tissue the resulting network should be able to produce this metabolite from simple precursors. More details can be found in the description of the method.

The output of our analysis is a cell type specific metabolic network for each of the cell types profiled in the HPA. As we are using HPA and gene expression data our networks do not represent the complete metabolic network that may be expressed in each cell type, but solely the part of the metabolic network that is expressed and hence the part of the network that is likely to be active. In order to provide a reliable and up to date genome-scale model template for our tissue/cell type specific metabolic networks, we first constructed the Human Metabolic Reaction (HMR) database containing the elements of previously published generic genome-scale human metabolic models [7], [8], [9] as well as the KEGG [23] database. This HMR database, which is publically available at www.metabolicatlas.com, will be periodically updated as new reactions are added to KEGG or MetaCyc or expression profiles for more proteins become available in HPA or other databases.

In order to evaluate the capability of our pipeline to generate reliable tissue specific metabolic networks, the metabolic model generated for hepatocytes was compared to HepatoNet1 [12], which is an extensively manually curated and annotated model of high quality. The availability of active metabolic networks corresponding to a broad set of healthy human cell types and cancers allows for a comparative analysis between cancer and healthy cell types in order to identify cancer specific metabolic features that constitute potential drug targets.

## Results/Discussion

### Database construction

The existing methods for the inference of tissue specific active metabolic networks have only used Recon1 as a scaffold. In order to integrate other sources of information we constructed the Human Metabolic Reaction database (HMR), containing the two existing genome-scale metabolic models, Recon1 and EHMN, as well as incorporating information from HumanCyc and KEGG.

The HMR database has a hierarchical structure in which the genes are at the top and are linked to information about their tissue specific expression profiles reported by Su et al [21] via BioGPS [24]. Each gene is linked to its different splicing variants and those to their corresponding proteins. Each protein is linked to its tissue specific abundances in the HPA database [18] and to the reactions they catalyze. The reactions are linked to metabolites that themselves are linked to their tissue specific information collected from the HMDB [22]. The HMR database will be regularly updated with new reactions contained in future genome-scale human metabolic reconstructions, as well as with new evidence included in future versions of the HPA, HMDB and newly published specific transcriptome data. Details regarding the construction and curation of the HMR database are available in the Methods section. The INIT algorithm requires a connected template human metabolic model as input, and this template model was generated from HMR. The template model contains 4,137 metabolites (3,397 unique) and 5,535 reactions (4,144 unique), which are associated to 1,512 metabolic genes. This template model is referred to as *iHuman1512*.

### Generation of 69 tissue specific and 16 cancer type specific genome-scale active metabolic networks

Using the INIT algorithm (see supplementary material for a detailed description), genome-scale active networks for 69 different cell types and 16 cancers were automatically generated. The resulting active metabolic networks are provided in SBML [25] format and are available at www.metabolicatlas.com.

The tissue specific models generated were compared with the BRENDA [26] collection of detected enzymes in various tissues. A hypergeometric test was carried out using the R statistical software. The reported p-values are the probabilities of obtaining an overlap higher than the observed with a random set of metabolic genes of the same size as the corresponding BRENDA entry. As it is shown in Table S1, all the comparisons between the models generated by our algorithm and BRENDA showed overlaps with p-values lower than 5e-4. Our computational liver model (*iHepatocyte1154*) shows a p-value of 1e-200, which is similar to the value obtained by comparing the manually reconstructed HepatoNet1 to BRENDA. 55% of the genes in *iHepatocyte1154* are also in BRENDA, while only 43% of the genes in HepatoNet1 are in BRENDA. The comparatively high p-values are for tissues for which there are very few annotated enzymes in BRENDA.

**Figure 3:**
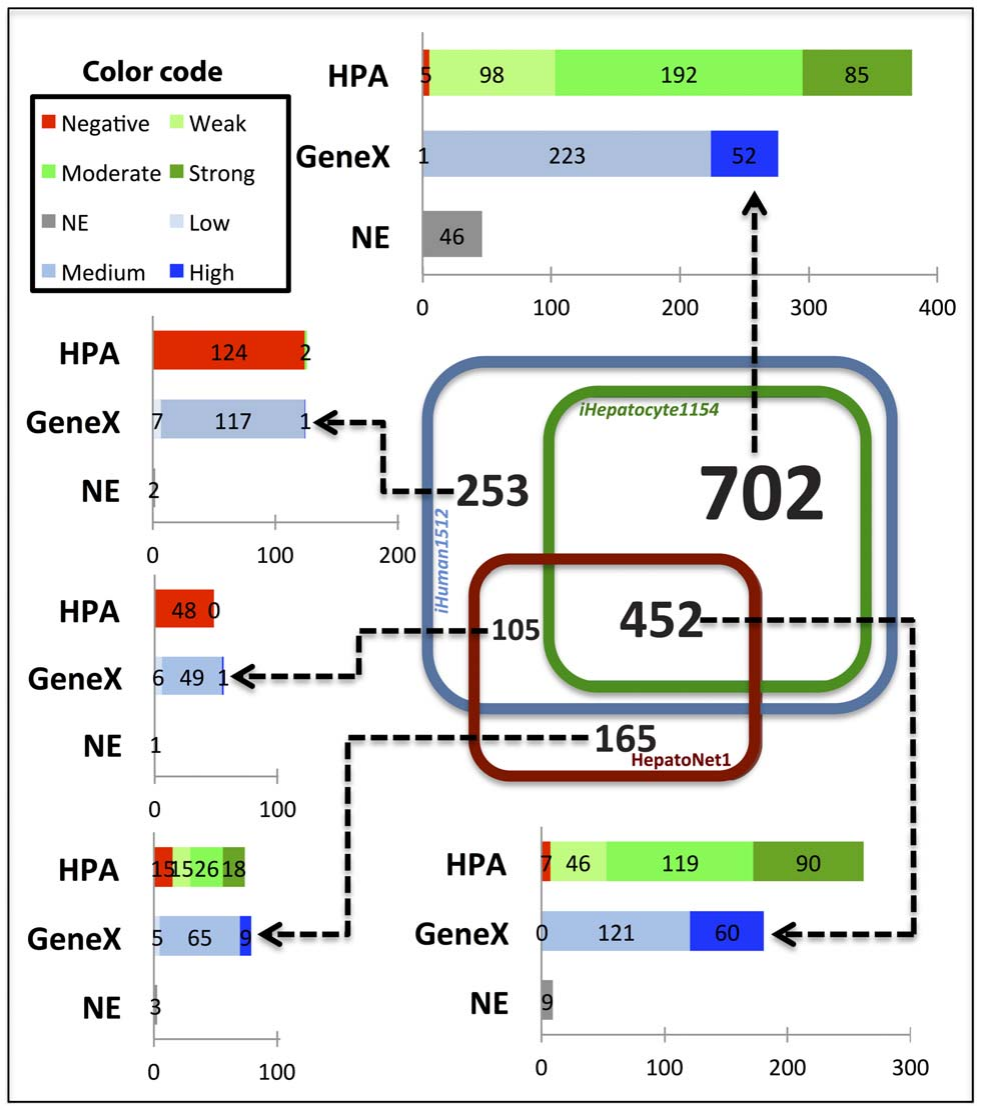
Gene content comparison between our hepatocyte model and HepatoNet1. The Venn diagram shows the overlap in terms of included genes between three models. The blue, green and red squares represent *iHuman1512*, our hepatocyte model *iHepatocyte1154* and HepatoNet1, respectively. The distribution of evidence scores of each section of the Venn diagram is plotted. The HPA evidence scores are illustrated as red, light, medium and dark green represent negative, weak moderate and strong expression, respectively. The transcriptome evidence scores (GeneX) are illustrated as red, light, medium and dark blue representing low, medium and high expression, respectively. No evidence (NE) is illustrated as grey color.

In order to validate the output of our algorithm, our automatically generated hepatocyte model was compared with HepatoNet1 [12], a manually curated and functional model of hepatocyte metabolism. The comparison was carried out at the gene level to avoid ambiguous decisions about reaction similarity. The overlap between the lists of genes included in each of the models is showed in Figure 3. Our hepatocyte model (*iHepatocyte1154*) contains 1,154 genes, of which 452 are also included in HepatoNet1 and 702 are absent. The evidence for the expression and translation of the 702 absent genes is as good as the evidence for the 452 genes that are in both networks, and we are therefore confident that the presence of most of the 702 extra genes has been correctly inferred by our algorithm. The HepatoNet1 network contains 261 genes not included in *iHepatocyte1154*, of which 156 were absent from our initial connected human network. Our algorithm could therefore not have assigned these genes to the hepatocyte sub-network and their existence reveals just a limitation of the data that were used as an input and not a limitation of our algorithm. 80 of these genes were not in HMR (see Table S2), but closer examination revealed that the majority (62 genes) of these genes corresponded to reactions that were actually present in HMR, but with different or absent gene associations. 13 out of the 18 remaining genes encode for transporters to the sinusoidal space; a type of blood vessels in the liver and therefore not a part of hepatocytes. The other 76 genes that were absent from *iHuman1512*, and their corresponding reactions, were removed because of being unbalanced, unconnected or otherwise problematic (see Table S3). 105 genes included in HepatoNet1, and present in *iHuman1512*, were not assigned by our algorithm to the hepatocyte-specific network. These genes correspond to 237 reactions, 132 of them still exist in *iHepatocyte1154* associated to different isoenzymes. The experimental evidences for the presence of these 105 genes (see Table S4) in the hepatocytes is mostly weak or negative, even slightly worse than the evidence for the 253 genes that were both rejected by our algorithm and absent in HepatoNet1, and we are therefore confident that these 105 genes were correctly rejected. This shows the importance of using cell type specific data when reconstructing GEMs, as enzyme isoforms can be differentially expressed in different cell types. Based on the above we can conclude that the mismatches between our hepatocyte-specific metabolic network and HepatoNet1 are accompanied by experimental evidence in favour of the choices made by our algorithm.

Finally we clustered the 69 plus 16 metabolic networks according to their similarity in terms of shared metabolic genes using unsupervised hierarchical clustering with average linkage and multiscale bootstrap resampling [27] (10,000 repetitions) implemented in the R statistical software (see Figure S1). The clustering shows, as it could be expected, local grouping of closely related cell types on the basis of cell anatomy (e.g. spleen in red pulp and white pulp cluster together). Interestingly, the cancers are separated into three different clusters, one containing liver, colorectal, breast and endometrial cancer, another minor cluster including cervical and head cancer and a third one containing the remaining ten cancers. It is also of interest to note that only 189 reactions (4.1% of the total number of reactions) are unique to a single cell or cancer type, while there is a larger core-set of 501 reactions (11.0% of total number of reactions) that are in common to all cells. Figure S2 shows the enrichment of some important metabolic pathways in the models.

### Identification of cancer specific metabolic features

Since the Warburg effect was observed at the beginning of the 20th century, it is known that cancer cells show characteristic metabolic features that make them different from healthy cells [28]. This supposed metabolic similarity between cancer cells justified the development of a generic cancer genome-scale metabolic model which was used to identify potential drug targets against cancer proliferation [29]. Here we have inferred active metabolic networks for 16 different cancer types, which can be compared with the 24 healthy cell types that they come from (there are several healthy cell types for some of the tissues associated to the cancers) in order to identify metabolic features that are characteristic of cancer. A hypergeometric test was used to identify genes and reactions that tend to be present in most of the cancer specific active metabolic networks and absent in most of the original healthy cell types (see Tables S5 and S6). The p-values obtained from the hypergeometric test were used to identify Reporter Metabolites [30] that are significantly more involved in the metabolism of cancer cells (see Table S7). The sets of genes, reactions and metabolites showing enrichment in the cancer active metabolic networks with p-values lower than 1e-4 are listed in the supplementary material. These lists of genes, reactions and metabolites are cancer specific features that are likely to be playing a specific role in proliferation of cancer cells and could be potential drug targets. Our comparative analysis between two sets of active metabolic networks can be seen as a high throughput hypothesis generation method. These hypotheses are not based on mere correlations between cancer and the presence of a particular protein, but being based on the underlying metabolic network structure, and hereby our analysis provides a mechanistic interpretation about the possible role of each identified feature on the proliferation of cancer.

One of the most significant results from the Reporter Metabolites analysis is a much more pronounced metabolism of polyamines (PAs) such as spermidine, spermine, and putrescine in cancer cells. PAs play a variety of roles, of which several are related to oxidative stress prevention and suppression of necrosis [31]. PAs have long been known to be of particular importance for rapidly proliferating cells, and as such its transport and synthesis have been thoroughly investigated as anti-cancer drug targets [32]. Inhibition of single enzymes in the PA synthesis pathway has proved disappointing, due to extensive regulation of the system and use of exogenous PAs by the cancer cells. Second generation drugs instead work by targeting the transport system, by structural homology to the PAs themselves, or by linking other aneoplastic drugs to the PAs [33].

Another high-ranking target is the isoprenoid biosynthesis pathway, in particular the intermediate geranylgeranyl diphosphate. This metabolite has been shown to promote oncogenic events due to its role in prenylation of important cancer proteins such as Ras and Rho GTPases [34]. Several drugs have therefore been developed to target the prenylation process [35] or the biosynthesis of geranylgeranyl diphosphate [36].

A third prominent group among the Reporter Metabolites is prostaglandins and leukotrienes together with the intermediate HPETE. These autocrine compounds are synthesized from arachidonic acid and are elevated in connection with inflammation. They have been shown to aid in cancer progression by promoting metastasis and by influencing the immune system [37]. Of particular interest is prostaglandin E2, where both the synthesis and degradation have been investigated as promising targets for drug development [38].

**Figure 4:**
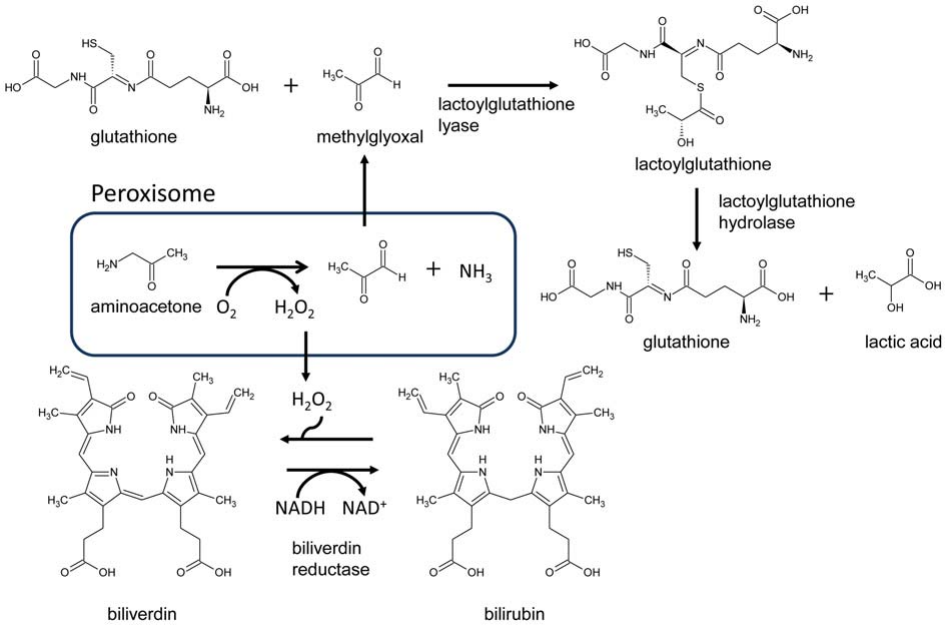
Example of a metabolic sub-network that was identified as being significantly more present in cancer tissues compared to their corresponding healthy tissues. Aminoacetone, which is a toxic by-product of amino acid catabolism, is converted to toxic methylglyoxal in a reaction that also result in hydrogen peroxide. The toxicity of methylglyoxal is relieved by two reaction steps involving ligation to glutathione and resulting in lactic acid. The generated hydrogen peroxide is taken care of by the enzyme biliverdin reductase. This is an example of how network-based analysis can lead to a more mechanistic interpretation of data.

The fact that so many of the identified targets correspond to well known and used drug targets, indicates that the method is able to generate biologically relevant hypotheses. Of particular interest are therefore the Reporter Metabolites that are currently not targeted in cancer treatment. Among the top-scoring Reporter Metabolites we identified biliverdin and bilirubin (Figure 4). Biliverdin reductase and the reactions catalyzed by this enzyme also appear among the genes and reaction most enriched in the cancer networks. Biliverdin reductase is known to be a major physiologic cytoprotectant against oxidative stress [39]. Cancer cells are known to be exposed to high oxidative stress resulting from the hydrogen peroxide generated during the oxidation of polyamines and other products of amino acid breakdown taking place in the peroxisome. Bilirubin is oxidized to biliverdin by hydrogen peroxide and subsequently reduced back to bilirubin by biliverdin reductase. This mechanism has been proven to be a major relief system for oxidative stress and could be considered a potential target against cancer proliferation. One of the hydrogen peroxide generating reactions taking place in the peroxisomes is the transformation of aminoacetone, which is an intermediate in the degradation of glycine, into methylglyoxal. Another source of methylglyoxal in cancer cells is from gluconeogenesis [40]. Methylglyoxal is known to be a toxic compound [41] that has been proven to induce apoptosis in some cancer cell lines [42]. Methylglyoxal also appeared among our top scoring reporter metabolites and both the gene coding for lactoylglutathione lyase (an enzyme that transforms methylglyoxal and glutathione into lactoylglutathione) and its associated reactions appear among the most enriched genes and reactions in the cancer active metabolic networks. Lactoylglutathione is further transformed into glutathione and lactic acid by the enzyme lactoylglutathione hydrolase (which also shows a significant enrichment in cancer metabolic networks with a p-value of 2e-3). Lactic acid is a well known metabolite produced by cancer cells. The mentioned two enzymes seem to be playing a relevant role in relieving the toxicity generated by methylglyoxal and could be potential drug targets against cancer proliferation. Targeting these enzymes would have the same effect on cancer cells as using methylglyoxal as a drug, but the advantage is that there would be no toxicity effects of methylglyoxal on healthy tissues.

### Conclusions and perspectives

We here present a method that is able to integrate different sources of biological evidence to generate high quality cell type specific metabolic networks. We used this method to generate genome-scale metabolic networks for 69 different human cell types and 16 cancer types, and this is the first step towards the establishment of a Human Metabolic Atlas, which may become a central portal for further advancing human metabolic models with the capability of performing tissue-level and organism-level metabolic simulations, allowing for a better understanding of complex diseases. The Human Metabolic Atlas and will be made publicly accessible for the medical and scientific community and may hereby become a valuable resource in the development of personalized medicine based on system-level analysis. An example of system-level analysis is the identification of cancer specific metabolic features that we have performed by comparing the networks generated using the INIT algorithm.

## Methods

### Database construction

In order to have an unambiguous characterization of metabolites and reactions, KEGG and InChI identifiers were used for standardization. Metabolites lacking identifiers to external databases were left out of the HMR database together with their corresponding reactions. The metabolite identifiers were used to infer if two reactions coming from different sources were the same. Each reaction was assigned to one or several of the eight compartments included in the HMR database: nucleus, cytosol, endoplasmatic reticulum, Golgi apparatus, peroxisomes, lysosomes, mitochondria and extracellular. In cases where the sub-cellular localization was absent from the template models it was inferred from immunohistochemical staining in the HPA. For enzymes that were not in the HPA, Swiss-Prot and GO were used to infer localization (see Table S8 for database versions). After removing the compounds that lack identifiers, the database contained 9,922 reactions, 2,366 genes, and 9,581 metabolites for the eight different compartments (3,547 unique metabolites and 6,319 unique reactions when compartmentalization is not considered). There are 338 of these metabolites which, even if they have KEGG identifiers, are generic compounds such as “Lipid” or “2-oxoacid”. Such compounds can lead to reactions that are elementally unbalanced. These problematic metabolites were removed, together with the 418 reactions in which they were involved after a detailed manual curation process. 38 reactions with wrong or unbalanced stoichiometries were also substituted by balanced versions during the curation process. In order to avoid problems associated with proton balancing, which arise from undefined protonation states of many metabolites, free exchange of protons was allowed in the models. Finally, all reactions unable to carry flux under any circumstance were removed. The reason for removing these unconnected reactions was that the algorithm requires a connected model as input. After this filtering, our template model contains 4,137 metabolites (3,397 unique) and 5,535 reactions (4,144 unique), which are associated to 1,512 metabolic genes (based on the Ensemble gene catalogue). This template model is referred to as *iHuman1512*. The numbers of genes maintained in each of the above mentioned steps are listed in Table S9. The discrepancy between the large number of reactions and the relatively small number of genes, which is also seen in previously published metabolic networks, is due to the fact that many reactions are included in the template networks based on literature studies or for connectivity reasons. In addition, some enzymes catalyze a large number of reactions and some enzymes catalyze reactions in several compartments. A comparison between *iHuman1512* and some published human metabolic networks is available in Table S10.

### Algorithm for the generation of tissue-specific models

Several algorithms aiming to obtain a tissue or condition-specific active set of metabolic reactions from a generic model have been previously developed. The first of these algorithms was the Gene Inactivity Moderated by Metabolism and Expression (GIMME) algorithm [43], which uses mRNA expression data as input. Two other algorithms were developed with the specific aim of generating human tissue specific metabolic networks. The first of those [10],_ENREF_10 developed by Shlomi and co-workers, used transcriptomic data as its sole input. The second one [11] was developed by the same authors in order to obtain a functional model for human hepatocytes and is able to integrate also metabolomic and proteomic data.

The INIT (Integrative Network Inference for Tissues) algorithm is formulated as a mixed integer-linear problem (MILP) and is specially tailored to use the evidence from the HPA as input. The problem is formulated so that all reactions in the resulting model are able to carry flux. The stoichiometric matrix S contains the stoichiometric coefficients for each internal metabolite in each reaction. By multiplying the stoichiometric matrix by the vector of reaction rates we obtain a vector of net accumulation or consumption rates for each internal metabolite. Instead of imposing the steady state condition for all the internal metabolites, as it is usually done, we allow for a small positive net accumulation rate. The net productions of metabolites will be given positive weights in the optimization. The reason for this choice is that we prefer to have a network able to synthesize molecules such as NADH or NADPH, rather than only being able to use them as cofactors. If a metabolite is present in a cell type (according to the HMDB) a positive net production of this metabolite will be imposed to the network in order to assure that all the reactions necessary for its synthesis are included in the tissue specific model.

Up to this date there is not a human biomass equation available in the literature (for example Recon1 incorporates a mouse biomass equation). On the other hand, human cells (with the exception of cancer cells), in contrast to microorganisms, do not tend to proliferate or do so slowly in comparison with the rest of their metabolic functions. This makes the biomass equation less relevant, unless the aim is to model cancer proliferation. Also human cells secrete into the blood a much broader spectrum of compounds than microbial cells secrete into their environment (which are mainly fermentation products). We therefore chose to generate networks allowing for secretion (or accumulation) of all their metabolites. If we had used the stricter steady state constraint, many reactions would have been removed from the models just because they were leading to dead end metabolites. These end metabolites could in fact be added to biomass or just be secreted into the blood stream, therefore we have aimed for a more flexible approach by allowing secretion (or net accumulation) of metabolites.

The MILP used in INIT can be specified as:
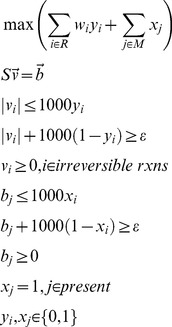
(1)The parameter ε is an arbitrarily small positive number. The weights of the binary variables corresponding to the reactions account for the evidence of their presence or absence. When the corresponding enzyme has been characterized in the HPA we have used values of *w_i_* of 20, 15, 10 and −8 for high, medium, low and absent proteins respectively. These scores are arbitrary and have been chosen to quantify the evidence colour codes that appear in the HPA. We have tested the sensitivity of the algorithm to the variation in these weights by perturbing them by 20% up and down and the impact on the output of the algorithm resulted only in small changes of the resulting networks. If the evidence comes from gene expression levels which were retrieved from BioGPS [24] and the publicly dataset “Human Body Index – Transcriptional Profiling” (GSE7307), we have used weights calculated as follows:
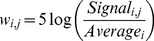
(2)The signal of gene *i* in tissue *j* is divided by the average signal across all the tissues. If the signal in a particular tissue is higher than its average across all the tissues the weight will be positive, if it is lower it will have a negative weight.

For the reactions that are related to several genes or proteins the highest evidence score is used. If no gene is associated to a particular reaction, or there is no proteomic or transcriptomic evidence, a weight of −2 is used in order to avoid adding unnecessary reactions without evidence and keep the network as parsimonious as possible. If a reaction linked to several genes is added to the final tissue specific network, only the genes showing a positive evidence score are kept in the tissue specific reaction-gene association.

The MILP problem was solved using MOSEK (www.mosek.com) and its Matlab interface.

## Supporting Information

Figure S1 **Clustering of 69 predicted cell type specific genome-scale metabolic models for normal tissues together with 16 for cancer tissues.** A dendrogram generated by unsupervised hierarchical clustering of the models based on predicted gene presence and absence is shown.
(PDF)

Figure S2 **The relative pathway enrichment profiles, based on KEGG pathways, for each of the models.** Blue corresponds to underrepresentation and red to overrepresentation. Note that it is the number of enzymes present for each pathway that underlie the comparison, not the abundances of the proteins.
(PDF)

Table S1 **Evaluation of the models by comparison to curated tissue-specific enzymes.** For each model, the set of genes is compared to the set of genes annotated as existing in the corresponding tissue in BRENDA. The p-values are derived from hypergeometric distribution.
(PDF)

Table S2 **Investigation of the 80 genes that were present in HepatoNet1 but missing in HMR.** The 80 missing genes were associated with 746 reactions in HepatoNet1, of which 597 metabolic and transport reactions were related to the sinusoidal space compartment. 117 metabolic reactions existed in HMR with different gene or no gene association and 32 (5 unique) reactions were altogether absent in HMR. KEGG reaction identifiers or Transporter Classification database identifiers (TCDB) are provided for the missing reactions.
(PDF)

Table S3 **Investigation of the 76 genes that were removed during the pre-processing steps.** 76 genes which were present in both HepatoNet1 and the HMR database were removed in order to get a fully connected input network for the INIT algorithm. This table summarizes which genes were removed during each of the pre-processing steps (see Table S2 for details).
(PDF)

Table S4 **Investigation of the 105 genes which are present in HepatoNet1 but missing in** ***iHepatocyte1154*** **due to the INIT algorithm.** The 105 missing genes were associated with 182 reactions in HepatoNet1, of which 5 metabolic reactions are related to the sinusoidal space compartment. 108 metabolic reactions existed in *iHepatocyte1154* with different gene or no gene association and 69 (60 unique) reactions are absent in *iHepatocyte1154* due to INIT algorithm. KEGG reaction identifiers are provided for the missing associated reactions to the genes.
(PDF)

Table S5 **List of reactions that were significantly more present in cancer tissues compared to their corresponding normal tissues (p-value<10e-4).**
(PDF)

Table S6 **List of genes for which their corresponding reactions were significantly more present in cancer tissues compared to their corresponding normal tissues (p-value<10e-4).**
(PDF)

Table S7 **List of Reporter Metabolites (p-value<10e-4).**
(PDF)

Table S8 **Versions of the databases used in the creation of the Human Metabolic Reaction database (HMR).**
(PDF)

Table S9 **Number of the genes after each pre-processing step during the generation of** ***iHuman1512*** **.** Since the INIT algorithm provides a connected and functional model, reactions that are unconnected in the template model can never be included. In order to separate between reactions that were excluded due to connectivity reasons and those that were excluded due to negative evidence, a number of preprocessing steps were performed on the data in the HMR database. In the first step reactions that contain very generic metabolites such as “lipid” or “alcohol” were removed. Reactions that were elementally unbalanced were fixed or removed. Simulations were performed to ensure that the network could not gain carbon, energy or redox power in an unbalanced manner. In the second step reactions where directionality information was lacking were removed. In the third step reactions where one or more of the substrates could not be synthesized through some other reaction (unconnected reactions) were removed. Finally, in the fourth step reactions that couldn't carry flux when the model had access to all exchange metabolites (as defined in the EHMN) were removed. Consequently, *iHuman1512*, a connected human network with 5,535 reactions, associated with 1512 protein coding genes, was generated. The number of genes associated to the remaining reactions after each pre-processing steps are presented below.
(PDF)

Table S10 **Comparison between iHuman1512 and some other published human metabolic networks.**
(PDF)
