# Supplementary material for: Reconstruction of Genome-Scale Active Metabolic Networks for 69 Human Cell Types and 16 Cancer Types Using INIT
Source: PLoS Comput Biol. 2012 May 17;8(5):e1002518. doi: 10.1371/journal.pcbi.1002518 (PMC3355067; doi:10.1371/journal.pcbi.1002518)
Supplement: Figure S2 — The relative pathway enrichment profiles, based on KEGG pathways, for each of the models. Blue corresponds to underrepresentation and red to overrepresentation. Note that it is the number of enzymes present for each pathway that underlie the comparison, not the abundances of the proteins. (PDF) [file pcbi.1002518.s002.pdf]

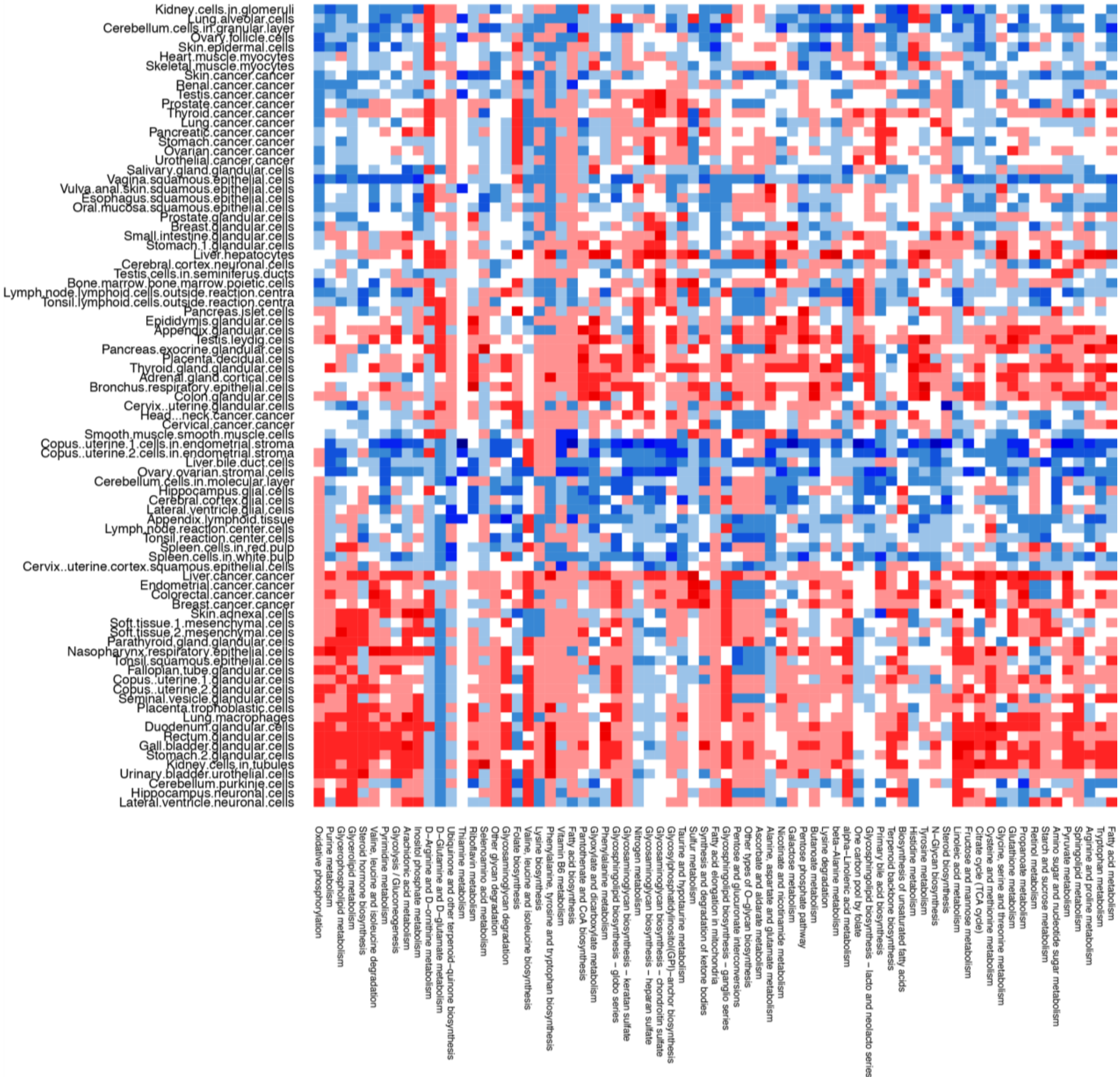


**Figure S2.** The relative pathway enrichment profiles, based on KEGG pathways, for each of the models. Blue corresponds to underrepresentation and red to overrepresentation. Note that it is the number of enzymes present for each pathway that underlie the comparison, not the abundances of the proteins.
