# Supplementary material for: Reconstruction of Genome-Scale Active Metabolic Networks for 69 Human Cell Types and 16 Cancer Types Using INIT
Source: PLoS Comput Biol. 2012 May 17;8(5):e1002518. doi: 10.1371/journal.pcbi.1002518 (PMC3355067; doi:10.1371/journal.pcbi.1002518)
Supplement: Table S1 — Evaluation of the models by comparison to curated tissue-specific enzymes. For each model, the set of genes is compared to the set of genes annotated as existing in the corresponding tissue in BRENDA. The p-values are derived from hypergeometric distribution. (PDF) [file pcbi.1002518.s003.pdf]

Table S1. Evaluation of the models by comparison to curated tissue-specific enzymes. For each model, the set of genes is compared to the set of genes annotated as existing in the corresponding tissue in BRENDA. The p-values are derived from hypergeometric distribution.

Model Cell type	BRENDA Tissue name	BRENDA EC numbers	BRENDA Ensembl	Model Ensembl	Overlap	p-value
Adrenal gland-cortical cells	adrenal gland	165	335	1113	169	9.82e-37
Bone marrow-bone marrow poietic cells	bone marrow	115	375	1031	168	7.23e-33
Breast-glandular cells	breast	56	259	1021	79	1.17e-06
Cerebellum-cells in granular layer	cerebellum	166	681	855	199	4.53e-22
Cerebral cortex-glial cells	cerebral cortex	122	542	965	180	1.85e-20
Colon-glandular cells	colon	196	590	1157	269	3.30e-45
Duodenum-glandular cells	duodenum	67	119	1202	74	1.92e-21
Epididymis-glandular cells	epididymis	84	241	1113	140	2.35e-39
Esophagus-squamous epithelial cells	esophagus	39	129	970	79	1.11e-28
Fallopian tube-glandular cells	fallopian tube	6	13	1154	10	2.58e-05
Gall bladder-glandular cells	gall bladder	14	40	1155	18	4.52e-04
Hippocampus-glial cells	hippocampus	147	635	962	200	6.59e-20
Kidney-cells in glomeruli	kidney	886	1521	826	491	1.53e-95
Liver-hepatocytes	liver	1484	1713	1154	820	1.0e-200
Lung-macrophages	lung	560	1140	1206	432	5.36e-43
Lymph node-lymphoid cells outside centra	lymph node	62	145	933	70	1.53e-18
Ovary-follicle cells	ovary	298	809	975	280	7.31e-36
Pancreas-exocrine glandular cells	pancreas	325	719	1112	287	1.82e-38
Placenta-decidual cells	placenta	347	806	1111	370	7.79e-72
Prostate-glandular cells	prostate	88	230	1036	103	5.25e-20
Rectum-glandular cells	rectum	18	38	1207	31	9.68e-15
Salivary gland-glandular cells	salivary gland	75	273	986	74	5.26e-05
Seminal vesicle-glandular cells	seminal vesicle	30	48	1172	26	5.67e-07
Skeletal muscle-myocytes	skeletal muscle	327	940	1041	357	2.16e-51
Skin-adnexal cells	skin	186	410	1181	246	4.07e-69
Small intestine-glandular cells	small intestine	260	557	1109	279	2.81e-62
Smooth muscle-smooth muscle cells	smooth muscle	52	183	996	79	1.72e-15
Spleen-cells in red pulp	spleen	471	941	1044	387	5.65e-68
Stomach 1-glandular cells	stomach	203	519	1077	220	1.11e-35
Testis-cells in seminiferus ducts	testis	445	1060	1045	414	1.50e-65
Thyroid gland-glandular cells	thyroid gland	84	326	1178	128	5.49e-14
Tonsil-lymphoid cells outside centra	tonsil	17	79	977	54	4.03e-23
Urinary bladder-urothelial cells	urinary bladder	20	29	1203	21	9.48e-09
