# Supplementary material for: Reconstruction of Genome-Scale Active Metabolic Networks for 69 Human Cell Types and 16 Cancer Types Using INIT
Source: PLoS Comput Biol. 2012 May 17;8(5):e1002518. doi: 10.1371/journal.pcbi.1002518 (PMC3355067; doi:10.1371/journal.pcbi.1002518)
Supplement: Table S2 — Investigation of the 80 genes that were present in HepatoNet1 but missing in HMR. The 80 missing genes were associated with 746 reactions in HepatoNet1, of which 597 metabolic and transport reactions were related to the sinusoidal space compartment. 117 metabolic reactions existed in HMR with different gene or no gene association and 32 (5 unique) reactions were altogether absent in HMR. KEGG reaction identifiers or Transporter Classification database identifiers (TCDB) are provided for the missing reactions. (PDF) [file pcbi.1002518.s004.pdf]

Table S2. Investigation of the 80 genes that were present in HepatoNet1 but missing in HMR. The 80 missing genes were associated with 746 reactions in HepatoNet1, of which 597 metabolic and transport reactions were related to the sinusoidal space compartment. 117 metabolic reactions existed in HMR with different gene or no gene association and 32 (5 unique) reactions were altogether absent in HMR. KEGG reaction identifiers or Transporter Classification database identifiers (TCDB) are provided for the missing reactions.

Color code	Explanation
	Liver specific sinusoidal space related reactions
	The associated reactions are missing from HMR
	The associated reactions are present in HMR, but under the control of other gene(s)
	The associated reactions are present in HMR, but with no associated genes
	The associated transport reactions are missing from HMR
	The associated transport reactions are present in HMR, but under the control of other gene(s)
	The associated transport reactions are present in HMR, but with no associated genes

Ensembl Gene ID	Score	Color Code
ENSG00000003987	-8,0000	
ENSG00000006534	3,1604	
ENSG00000012660	15,0000	
ENSG00000021488	0,0205	
ENSG00000054148	15,0000	
ENSG00000054179	15,0000	
ENSG00000063601	10,0000	
ENSG00000072778	15,0000	
ENSG00000087053	0,7663	
ENSG00000099797	15,0000	KEGG ID:R07765
ENSG00000100075	15,0000	TCDB:2.A.29.7.2
ENSG00000101986	4,4575	
ENSG00000102125	0,0232	
ENSG00000103064	0,8452	
ENSG00000103257	-5,4312	
ENSG00000108528	15,0000	
ENSG00000110911	1,1849	
ENSG00000111775	0,0000	
ENSG00000112303	-3,9297	
ENSG00000112695	0,8719	
ENSG00000112981	-0,8115	

ENSG00000121053	0,2721	
ENSG00000123684	15,0000	
ENSG00000125454	-0,0169	
ENSG00000125505	1,4716	
ENSG00000126267	20,0000	
ENSG00000127184	-1,4591	
ENSG00000127540	8,0144	
ENSG00000131143	15,0000	
ENSG00000131174	-0,1160	
ENSG00000135220	0,0128	
ENSG00000135940	20,0000	
ENSG00000139505	-0,0429	
ENSG00000140598	0,0292	
ENSG00000140740	15,0000	
ENSG00000141446	0,1746	
ENSG00000142046	0,0045	
ENSG00000152463	20,0000	
ENSG00000152642	15,0000	
ENSG00000155380	15,0000	
ENSG00000156467	0,6927	
ENSG00000156508	-0,1603	
ENSG00000156885	-3,0846	
ENSG00000156973	0,2023	
ENSG00000158296	15,0000	
ENSG00000158865	-8,0000	
ENSG00000160190	15,0000	
ENSG00000160471	10,0000	
ENSG00000161281	-3,3511	
ENSG00000164919	20,0000	
ENSG00000165029	0,8731	
ENSG00000165996	-0,4397	KEGG ID:R07764
ENSG00000167325	-8,0000	
ENSG00000167658	10,0000	
ENSG00000169021	6,0894	
ENSG00000169100	-0,9951	
ENSG00000169359	0,7316	
ENSG00000170385	10,0000	
ENSG00000170516	-0,0085	
ENSG00000171320	0,0329	
ENSG00000171848	-4,4222	
ENSG00000172197	0,0000	
ENSG00000173610	0,6157	
ENSG00000173660	-2,2642	
ENSG00000176340	6,4774	
ENSG00000177646	0,0148	

ENSG00000178537	20,0000	TCDB:2.A.29.8.3
ENSG00000178741	15,0000	
ENSG00000184076	3,3348	
ENSG00000184210	10,0000	
ENSG00000185633	-0,1550	
ENSG00000186792	-0,1046	
ENSG00000187581	-8,0000	
ENSG00000187758	20,0000	
ENSG00000188687	0,0961	
ENSG00000196517	10,0000	
ENSG00000197977	0,3847	
ENSG00000206527	10,0000	KEGG ID:R07764
ENSG00000215151	0,4151	
ENSG00000221988	2,1999	
