# Supplementary material for: Reconstruction of Genome-Scale Active Metabolic Networks for 69 Human Cell Types and 16 Cancer Types Using INIT
Source: PLoS Comput Biol. 2012 May 17;8(5):e1002518. doi: 10.1371/journal.pcbi.1002518 (PMC3355067; doi:10.1371/journal.pcbi.1002518)
Supplement: Table S3 — Investigation of the 76 genes that were removed during the pre-processing steps. 76 genes which were present in both HepatoNet1 and the HMR database were removed in order to get a fully connected input network for the INIT algorithm. This table summarizes which genes were removed during each of the pre-processing steps (see Table S2 for details). (PDF) [file pcbi.1002518.s005.pdf]

Table S3. Investigation of the 76 genes that were removed during the pre-processing steps. 76 genes which were present in both HepatoNet1 and the HMR database were removed in order to get a fully connected input network for the INIT algorithm. This table summarizes which genes were removed during each of the pre-processing steps (see Table S2 for details).

Ensembl Gene ID	Score	HMR	Step 1	Step 2	Step 3	Step 4
ENSG00000002549	20,0000	✓	✓	N/A	N/A	N/A
ENSG00000005469	20,0000	✓	✓	✓	N/A	N/A
ENSG00000010256	20,0000	✓	✓	N/A	N/A	N/A
ENSG00000065989	20,0000	✓	N/A	N/A	N/A	N/A
ENSG00000072756	-0,0479	✓	N/A	N/A	N/A	N/A
ENSG00000073417	15,0000	✓	✓	N/A	N/A	N/A
ENSG00000074410	10,0000	✓	✓	✓	N/A	N/A
ENSG00000095321	10,0000	✓	✓	✓	✓	N/A
ENSG00000100092	-8,0000	✓	✓	N/A	N/A	N/A
ENSG00000100294	1,0765	✓	✓	N/A	N/A	N/A
ENSG00000100354	-8,0000	✓	✓	N/A	N/A	N/A
ENSG00000102032	-8,0000	✓	✓	✓	N/A	N/A
ENSG00000102575	-8,0000	✓	✓	✓	N/A	N/A
ENSG00000104267	15,0000	✓	✓	✓	N/A	N/A
ENSG00000105650	0,2029	✓	N/A	N/A	N/A	N/A
ENSG00000107159	-8,0000	✓	✓	✓	N/A	N/A
ENSG00000111684	0,6493	✓	✓	✓	N/A	N/A
ENSG00000111726	-0,1097	✓	✓	✓	✓	N/A
ENSG00000113231	-0,0805	✓	N/A	N/A	N/A	N/A
ENSG00000113448	0,4340	✓	N/A	N/A	N/A	N/A
ENSG00000114021	1,7586	✓	✓	N/A	N/A	N/A
ENSG00000114054	1,7888	✓	✓	✓	✓	N/A
ENSG00000115252	20,0000	✓	✓	N/A	N/A	N/A
ENSG00000115944	1,6733	✓	✓	N/A	N/A	N/A
ENSG00000118094	0,2086	✓	✓	✓	N/A	N/A
ENSG00000118298	15,0000	✓	✓	✓	N/A	N/A
ENSG00000120437	20,0000	✓	✓	N/A	N/A	N/A
ENSG00000123360	10,0000	✓	✓	N/A	N/A	N/A
ENSG00000124713	20,0000	✓	✓	N/A	N/A	N/A
ENSG00000125877	15,0000	✓	✓	N/A	N/A	N/A
ENSG00000131055	-8,0000	✓	✓	N/A	N/A	N/A
ENSG00000131238	20,0000	✓	✓	N/A	N/A	N/A
ENSG00000131686	-8,0000	✓	✓	✓	N/A	N/A
ENSG00000132746	0,3473	✓	N/A	N/A	N/A	N/A
ENSG00000133256	1,0051	✓	N/A	N/A	N/A	N/A
ENSG00000133742	15,0000	✓	✓	✓	N/A	N/A
ENSG00000134575	11,9207	✓	✓	✓	N/A	N/A

ENSG00000138030	20,0000	✓	✓	✓	N/A	N/A
ENSG00000138356	3,0597	✓	✓	✓	N/A	N/A
ENSG00000138735	15,0000	✓	N/A	N/A	N/A	N/A
ENSG00000139053	-4,7002	✓	N/A	N/A	N/A	N/A
ENSG00000139684	4,6124	✓	✓	✓	✓	N/A
ENSG00000142513	0,0781	✓	N/A	N/A	N/A	N/A
ENSG00000145284	-0,2561	✓	✓	✓	N/A	N/A
ENSG00000152270	15,0000	✓	✓	N/A	N/A	N/A
ENSG00000154678	-0,0267	✓	N/A	N/A	N/A	N/A
ENSG00000157353	0,0536	✓	✓	✓	N/A	N/A
ENSG00000159348	20,0000	✓	✓	N/A	N/A	N/A
ENSG00000162551	20,0000	✓	✓	✓	N/A	N/A
ENSG00000163283	-4,3278	✓	✓	✓	N/A	N/A
ENSG00000163286	-8,0000	✓	✓	✓	N/A	N/A
ENSG00000163295	0,1013	✓	✓	✓	N/A	N/A
ENSG00000164405	9,3504	✓	✓	N/A	N/A	N/A
ENSG00000164879	-8,0000	✓	✓	✓	N/A	N/A
ENSG00000167434	10,0000	✓	✓	✓	N/A	N/A
ENSG00000168748	0,7054	✓	✓	✓	N/A	N/A
ENSG00000169239	20,0000	✓	✓	✓	N/A	N/A
ENSG00000171298	1,7621	✓	✓	✓	N/A	N/A
ENSG00000171408	10,0000	✓	N/A	N/A	N/A	N/A
ENSG00000172572	-8,0000	✓	N/A	N/A	N/A	N/A
ENSG00000172955	13,3880	✓	N/A	N/A	N/A	N/A
ENSG00000173599	4,3108	✓	✓	✓	✓	N/A
ENSG00000174990	0,0000	✓	✓	✓	N/A	N/A
ENSG00000175198	0,8342	✓	✓	✓	✓	N/A
ENSG00000178538	10,0000	✓	✓	✓	N/A	N/A
ENSG00000179091	15,0000	✓	✓	N/A	N/A	N/A
ENSG00000181915	0,6908	✓	✓	✓	✓	N/A
ENSG00000183828	-0,0390	✓	N/A	N/A	N/A	N/A
ENSG00000184254	-2,1638	✓	N/A	N/A	N/A	N/A
ENSG00000184588	-8,0000	✓	N/A	N/A	N/A	N/A
ENSG00000185527	0,1613	✓	N/A	N/A	N/A	N/A
ENSG00000186642	15,0000	✓	N/A	N/A	N/A	N/A
ENSG00000198099	15,0000	✓	✓	N/A	N/A	N/A
ENSG00000205268	10,0000	✓	✓	N/A	N/A	N/A
ENSG00000205560	10,0000	✓	✓	✓	N/A	N/A
ENSG00000214013	15,0000	✓	✓	✓	✓	N/A

✓ : Available, N/A: Not Available
