# Supplementary material for: Reconstruction of Genome-Scale Active Metabolic Networks for 69 Human Cell Types and 16 Cancer Types Using INIT
Source: PLoS Comput Biol. 2012 May 17;8(5):e1002518. doi: 10.1371/journal.pcbi.1002518 (PMC3355067; doi:10.1371/journal.pcbi.1002518)
Supplement: Table S4 — Investigation of the 105 genes which are present in HepatoNet1 but missing in iHepatocyte1154 due to the INIT algorithm. The 105 missing genes were associated with 182 reactions in HepatoNet1, of which 5 metabolic reactions are related to the sinusoidal space compartment. 108 metabolic reactions existed in iHepatocyte1154 with different gene or no gene association and 69 (60 unique) reactions are absent in iHepatocyte1154 due to INIT algorithm. KEGG reaction identifiers are provided for the missing associated reactions to the genes. (PDF) [file pcbi.1002518.s006.pdf]

Table S4. Investigation of the 105 genes which are present in HepatoNet1 but missing in *iHepatocyte1154* due to the INIT algorithm. The 105 missing genes were associated with 182 reactions in HepatoNet1, of which 5 metabolic reactions are related to the sinusoidal space compartment. 108 metabolic reactions existed in *iHepatocyte1154* with different gene or no gene association and 69 (60 unique) reactions are absent in *iHepatocyte1154* due to INIT algorithm. KEGG reaction identifiers are provided for the missing associated reactions to the genes.

Color code	Explanation
(yellow)	Liver specific sinusoidal space related reactions
(red)	The associated reactions are present in HMR, but absent in *iHepatocyte1154* due to the INIT algorithm
(green)	At least one of the associated reaction is present in *iHepatocyte1154*, but under the control of other gene(s)

Ensembl Gene ID	Color Code
ENSG00000001630	R05640 (red)
ENSG00000002726	(green)
ENSG00000005381	(green)
ENSG00000007350	R01067, R01641 (red)
ENSG00000014257	(green)
ENSG00000026652	R07762 (red)
ENSG00000038274	(green)
ENSG00000049860	R00022 (red)
ENSG00000062485	R00351 (red)
ENSG00000065154	R00667 (red)
ENSG00000065911	(green)
ENSG00000067057	R00769, R01843, R03237, R03239 (green)
ENSG00000067064	(green)
ENSG00000067225	R00200, R00430, R00572, R00659, R01138, R01858 (red)
ENSG00000070669	(green)
ENSG00000078070	R04138 (red)
ENSG00000081760	R01357, R01357 (red)
ENSG00000089250	R00111, R00557, R00558 (red)
ENSG00000097021	(green)
ENSG00000100288	R01021, R01468 (red)
ENSG00000100596	(green)
ENSG00000101210	(green)
ENSG00000101911	R01049 (red)
ENSG00000102144	R01512 (red)
ENSG00000102172	(green)
ENSG00000103024	(green)

ENSG00000105679	R01061
ENSG00000106348	
ENSG00000109107	
ENSG00000110717	R02163
ENSG00000111674	
ENSG00000111716	R00703
ENSG00000111732	
ENSG00000112972	
ENSG00000113552	R00765
ENSG00000115159	
ENSG00000116981	
ENSG00000119421	R02163
ENSG00000120137	R02971, R03018, R04391
ENSG00000121281	
ENSG00000122643	
ENSG00000125356	R02163
ENSG00000125779	R02971, R03018, R04391
ENSG00000128683	
ENSG00000131459	
ENSG00000131844	R04138
ENSG00000132518	
ENSG00000133731	
ENSG00000136143	R00405
ENSG00000138801	R00509
ENSG00000139163	R01468
ENSG00000139180	R02163
ENSG00000140057	
ENSG00000140287	R01167
ENSG00000141401	
ENSG00000141934	R06520
ENSG00000143179	R00513, R02372, R00964, R02332
ENSG00000143199	
ENSG00000143627	R00200, R00430, R00572, R00659, R01138, R01858, R01010
ENSG00000143727	
ENSG00000143845	R01468
ENSG00000144362	R00173
ENSG00000147224	R01049
ENSG00000147684	R02163
ENSG00000149925	
ENSG00000151376	
ENSG00000152254	
ENSG00000152782	R02971, R03018, R04391
ENSG00000153574	

ENSG00000154930	R00235, R00236, R00316, R00925, R00926, R01354
ENSG00000160191	
ENSG00000160209	R00174
ENSG00000160211	
ENSG00000163082	R06520
ENSG00000163114	
ENSG00000163281	R00765
ENSG00000163655	R01230, R01231
ENSG00000163738	
ENSG00000164039	
ENSG00000164708	R01518
ENSG00000164867	R00111, R00557, R00558
ENSG00000166796	R00703
ENSG00000167419	
ENSG00000168032	
ENSG00000168393	R02094, R02098
ENSG00000168653	R02163
ENSG00000168710	
ENSG00000170950	R01512
ENSG00000171989	R00703
ENSG00000172340	R00405
ENSG00000172954	R07762
ENSG00000173175	
ENSG00000176153	
ENSG00000176454	R07762
ENSG00000179087	R00028, R00801
ENSG00000182054	
ENSG00000183696	
ENSG00000184752	R02163
ENSG00000189043	R02163
ENSG00000196475	
ENSG00000197217	
ENSG00000197594	R00160, R03036, R00287
ENSG00000198610	R04819, R04825
ENSG00000198931	R00190, R01229, R00190
ENSG00000229937	R01049
