# Supplementary material for: Reconstruction of Genome-Scale Active Metabolic Networks for 69 Human Cell Types and 16 Cancer Types Using INIT
Source: PLoS Comput Biol. 2012 May 17;8(5):e1002518. doi: 10.1371/journal.pcbi.1002518 (PMC3355067; doi:10.1371/journal.pcbi.1002518)
Supplement: Table S5 — List of reactions that were significantly more present in cancer tissues compared to their corresponding normal tissues (p-value<10e-4). (PDF) [file pcbi.1002518.s007.pdf]

Table S5. List of reactions that were significantly more present in cancer tissues compared to their corresponding normal tissues (p-value < 10e-4).

Reactions with reactants and products
ATP[mitochondria] + CoA[mitochondria] + Succinate[mitochondria] <=> ADP[mitochondria] + Succinyl-CoA[mitochondria] + Orthophosphate[mitochondria]
NAD+[cytosol] + L-Arginine[cytosol] <=> N(omega)-(ADP-D-ribosyl)-L-arginine[cytosol] + Nicotinamide[cytosol]
L-Tyrosine[peroxisomes] => Tyramine[peroxisomes] + CO2[peroxisomes]
NAD+[cytosol] + sn-Glycerol 3-phosphate[cytosol] <=> NADH[cytosol] + Glycerone phosphate[cytosol] + H+[cytosol]
NADP+[endoplasmic reticulum] + Cholesterol[endoplasmic reticulum] <=> NADPH[endoplasmic reticulum] + Cholesta-5,7-dien-3beta-ol[endoplasmic reticulum] + H+[endoplasmic reticulum]
1H-Imidazole-4-ethanamine[extracellular] + H2O[extracellular] + Oxygen[extracellular] => Imidazole-4-acetaldehyde[extracellular] + H2O2[extracellular] + NH3[extracellular]
Tyramine[peroxisomes] + H2O[peroxisomes] + Oxygen[peroxisomes] => 4-Hydroxyphenylacetaldehyde[peroxisomes] + H2O2[peroxisomes] + NH3[peroxisomes]
Phenethylamine[peroxisomes] + H2O[peroxisomes] + Oxygen[peroxisomes] => Phenylacetaldehyde[peroxisomes] + H2O2[peroxisomes] + NH3[peroxisomes]
1-Acyl-sn-glycero-3-phosphocholine[extracellular] + H2O[extracellular] => sn-glycero-3-Phosphocholine[extracellular] + Fatty acid[extracellular]
2-Acyl-sn-glycero-3-phosphocholine[extracellular] + H2O[extracellular] => sn-glycero-3-Phosphocholine[extracellular] + Fatty acid[extracellular]
1,3-Diaminopropane[extracellular] + H2O[extracellular] + Oxygen[extracellular] => 3-Aminopropanal[extracellular] + H2O2[extracellular] + NH3[extracellular]
1-Acyl-sn-glycero-3-phosphoethanolamine[extracellular] + H2O[extracellular] => sn-glycero-3-Phosphoethanolamine[extracellular] + Fatty acid[extracellular]
2-Acyl-sn-glycero-3-phosphoethanolamine[extracellular] + H2O[extracellular] => sn-glycero-3-Phosphoethanolamine[extracellular] + Fatty acid[extracellular]
ATP[nucleus] + D-myo-Inositol 1,4,5-trisphosphate[nucleus] => ADP[nucleus] + 1D-myo-Inositol 1,3,4,5-tetrakisphosphate[nucleus]
N-Methylputrescine[extracellular] + Oxygen[extracellular] => 1-Methylpyrrolinium[extracellular] + H2O2[extracellular] + NH3[extracellular]
N-Acetylmuramoyl-Ala[extracellular] + H2O[extracellular] => N-Acetyl-D-muramoate[extracellular] + L-Alanine[extracellular]
Dopamine[peroxisomes] + H2O[peroxisomes] + Oxygen[peroxisomes] => 3,4-Dihydroxyphenylacetaldehyde[peroxisomes] + H2O2[peroxisomes] + NH3[peroxisomes]
D-myo-Inositol 3,4-bisphosphate[cytosol] + H2O[cytosol] => 1D-myo-Inositol 3-phosphate[cytosol] + Orthophosphate[cytosol]
2-Acetyl-1-alkyl-sn-glycero-3-phosphocholine[extracellular] + H2O[extracellular] => Acetate[extracellular] + 1-Organyl-2-lyso-sn-glycero-3-phosphocholine[extracellular]
Reduced riboflavin[cytosol] + NADP+[cytosol] <=> Riboflavin[cytosol] + NADPH[cytosol] + H+[cytosol]
Cadaverine[extracellular] + H2O[extracellular] + Oxygen[extracellular] => 5-Aminopentanal[extracellular] + H2O2[extracellular] + NH3[extracellular]

Spermine[extracellular] + H2O[extracellular] + Oxygen[extracellular] => spermine monoaldehyde[extracellular] + H2O2[extracellular] + NH4+[extracellular]
Spermidine[extracellular] + H2O[extracellular] + Oxygen[extracellular] => spermidine monoaldehyde 2[extracellular] + H+[extracellular] + H2O2[extracellular] + NH4+[extracellular]
spermidine monoaldehyde 2[extracellular] + H+[extracellular] + H2O[extracellular] + Oxygen[extracellular] => spermidine dialdehyde[extracellular] + H2O2[extracellular] + NH4+[extracellular]
spermine monoaldehyde[extracellular] + H2O[extracellular] + Oxygen[extracellular] => spermine dialdehyde[extracellular] + H2O2[extracellular] + NH4+[extracellular]
Spermidine[extracellular] + H2O[extracellular] + Oxygen[extracellular] => spermidine monoaldehyde 1[extracellular] + H2O2[extracellular] + NH4+[extracellular]
trans,cis-Lauro-2,6-dienoyl-CoA[peroxisomes] + H2O[peroxisomes] <=> (3S)-3-hydroxydodec-cis-6-enoyl-CoA[peroxisomes]
NADPH[nucleus] + cholesta-5,7,24-trien-3beta-ol[nucleus] + H+[nucleus] <=> NADP+[nucleus] + Desmosterol[nucleus]
Reduced FMN[cytosol] + NADP+[cytosol] <=> FMN[cytosol] + NADPH[cytosol] + H+[cytosol]
Dolichyl phosphate D-mannose[endoplasmic reticulum] + mannosealpha1-6(ethanolaminephosphate-2)mannosealpha1-4(acyl)glucosaminylphosphatidylinositol[endoplasmic reticulum] <=> Dolichyl phosphate[endoplasmic reticulum] + mannosealpha1-2mannosealpha1-6(ethanolaminephosphate-2)mannosealpha1-4glucosaminyl-acyl-phosphatidylinositol[endoplasmic reticulum]
(2S,6R,10R)-trimethyl-2E-hendecenoyl-CoA[peroxisomes] + H2O[peroxisomes] <=> 3(R)-hydroxy-(2S,6R,10)-trimethyl-hendecanoyl-CoA[peroxisomes]
1-acyl-2-lyso-phosphatidylserine[extracellular] + H2O[extracellular] <=> glycerophosphoserine[extracellular] + Carboxylate[extracellular] + H+[extracellular]
5-oxo-12(S)-hydroxy-eicosa-2E,8E,10E,14Z-tetraenoyl-CoA[peroxisomes] + H2O[peroxisomes] <=> 3(S),12(S)-dihydroxy-5-oxo-eicosa-8E,10E,14Z-trienoyl-CoA[peroxisomes]
spermidine monoaldehyde 1[extracellular] + H2O[extracellular] + Oxygen[extracellular] => spermidine dialdehyde[extracellular] + H2O2[extracellular] + NH4+[extracellular]
timnodonate[peroxisomes] + Oxygen[peroxisomes] => 15(R)-hydroperoxy-EPE[peroxisomes]
prostaglandin H1[nucleus] <=> Alprostadil[nucleus]
L-Alanine[cytosol] + H+[cytosol] <=> L-Alanine[mitochondria] + H+[mitochondria]
Phenethylamine[cytosol] => Phenethylamine[peroxisomes]
timnodonate[cytosol] <=> timnodonate[peroxisomes]
Bilirubin[extracellular] + HCO3-[cytosol] <=> Bilirubin[cytosol] + HCO3-[extracellular]
ATP[cytosol] + D-myo-Inositol 1,4,5-trisphosphate[cytosol] => ADP[cytosol] + 1D-myo-Inositol 1,3,4,5-tetrakisphosphate[cytosol] + 2 H+[cytosol]
1-Phosphatidyl-1D-myo-inositol 4-phosphate[cytosol] + H2O[cytosol] => 6-(alpha-D-Glucosaminyl)-1-phosphatidyl-1D-myo-inositol[cytosol] + Orthophosphate[cytosol]
ATP[cytosol] + 5-PP-InsP5[cytosol] => ADP[cytosol] + 3,5-bisdiphosphoinositol-1D-myo-inositol (2,3,4,6)tetrakisphosphate[cytosol] + H+[cytosol]
Glutathione[cytosol] + Methylglyoxal[cytosol] => (R)-S-Lactoylglutathione[cytosol]
NADH[cytosol] + Biliverdin[cytosol] => NAD+[cytosol] + Bilirubin[cytosol]
NADPH[cytosol] + Biliverdin[cytosol] => NADP+[cytosol] + Bilirubin[cytosol]
DL-Glyceraldehyde 3-phosphate[cytosol] + Sedoheptulose 7-phosphate[cytosol] => D-

Erythrose 4-phosphate[cytosol] + D-Fructose 6-phosphate[cytosol]
Reduced riboflavin[cytosol] + NADP+[cytosol] => Riboflavin[cytosol] + NADPH[cytosol] + 2 H+[cytosol]
NAD+[cytosol] + sn-Glycerol 3-phosphate[cytosol] <=> NADH[cytosol] + Glycerone phosphate[cytosol] + H+[cytosol]
Isopentenyl diphosphate[cytosol] => Diphosphate[cytosol]
all-trans-Hexaprenyl diphosphate[cytosol] + Isopentenyl diphosphate[cytosol] => all-trans-Heptaprenyl diphosphate[cytosol] + Diphosphate[cytosol]
all-trans-Pentaprenyl diphosphate[cytosol] + Isopentenyl diphosphate[cytosol] => all-trans-Heptaprenyl diphosphate[cytosol] + Diphosphate[cytosol]
di-trans,poly-cis-Decaprenyl diphosphate[cytosol] + Isopentenyl diphosphate[cytosol] => all-trans-Heptaprenyl diphosphate[cytosol] + Diphosphate[cytosol]
trans,trans-Farnesyl diphosphate[cytosol] + Isopentenyl diphosphate[cytosol] => all-trans-Heptaprenyl diphosphate[cytosol] + Diphosphate[cytosol]
Geranylgeranyl diphosphate[cytosol] + Isopentenyl diphosphate[cytosol] => all-trans-Heptaprenyl diphosphate[cytosol] + Diphosphate[cytosol]
all-trans-Octaprenyl diphosphate[cytosol] + Isopentenyl diphosphate[cytosol] => all-trans-Heptaprenyl diphosphate[cytosol] + Diphosphate[cytosol]
all-trans-Nonaprenyl diphosphate[cytosol] + Isopentenyl diphosphate[cytosol] => all-trans-Heptaprenyl diphosphate[cytosol] + Diphosphate[cytosol]
all-trans-Heptaprenyl diphosphate[cytosol] + Isopentenyl diphosphate[cytosol] => all-trans-Hexaprenyl diphosphate[cytosol] + Diphosphate[cytosol]
Isopentenyl diphosphate[cytosol] => Diphosphate[cytosol]
all-trans-Pentaprenyl diphosphate[cytosol] + Isopentenyl diphosphate[cytosol] => all-trans-Hexaprenyl diphosphate[cytosol] + Diphosphate[cytosol]
di-trans,poly-cis-Decaprenyl diphosphate[cytosol] + Isopentenyl diphosphate[cytosol] => all-trans-Hexaprenyl diphosphate[cytosol] + Diphosphate[cytosol]
trans,trans-Farnesyl diphosphate[cytosol] + Isopentenyl diphosphate[cytosol] => all-trans-Hexaprenyl diphosphate[cytosol] + Diphosphate[cytosol]
Geranylgeranyl diphosphate[cytosol] + Isopentenyl diphosphate[cytosol] => all-trans-Hexaprenyl diphosphate[cytosol] + Diphosphate[cytosol]
all-trans-Octaprenyl diphosphate[cytosol] + Isopentenyl diphosphate[cytosol] => all-trans-Hexaprenyl diphosphate[cytosol] + Diphosphate[cytosol]
all-trans-Nonaprenyl diphosphate[cytosol] + Isopentenyl diphosphate[cytosol] => all-trans-Hexaprenyl diphosphate[cytosol] + Diphosphate[cytosol]
all-trans-Heptaprenyl diphosphate[cytosol] + Isopentenyl diphosphate[cytosol] => all-trans-Pentaprenyl diphosphate[cytosol] + Diphosphate[cytosol]
all-trans-Hexaprenyl diphosphate[cytosol] + Isopentenyl diphosphate[cytosol] => all-trans-Pentaprenyl diphosphate[cytosol] + Diphosphate[cytosol]
Isopentenyl diphosphate[cytosol] => Diphosphate[cytosol]
di-trans,poly-cis-Decaprenyl diphosphate[cytosol] + Isopentenyl diphosphate[cytosol] => all-trans-Pentaprenyl diphosphate[cytosol] + Diphosphate[cytosol]
trans,trans-Farnesyl diphosphate[cytosol] + Isopentenyl diphosphate[cytosol] => all-trans-Pentaprenyl diphosphate[cytosol] + Diphosphate[cytosol]
all-trans-Octaprenyl diphosphate[cytosol] + Isopentenyl diphosphate[cytosol] => all-trans-Pentaprenyl diphosphate[cytosol] + Diphosphate[cytosol]

all-trans-Nonaprenyl diphosphate[cytosol] + Isopentenyl diphosphate[cytosol] => all-trans-Pentaprenyl diphosphate[cytosol] + Diphosphate[cytosol]
all-trans-Heptaprenyl diphosphate[cytosol] + Isopentenyl diphosphate[cytosol] => di-trans,poly-cis-Decaprenyl diphosphate[cytosol] + Diphosphate[cytosol]
all-trans-Hexaprenyl diphosphate[cytosol] + Isopentenyl diphosphate[cytosol] => di-trans,poly-cis-Decaprenyl diphosphate[cytosol] + Diphosphate[cytosol]
all-trans-Pentaprenyl diphosphate[cytosol] + Isopentenyl diphosphate[cytosol] => di-trans,poly-cis-Decaprenyl diphosphate[cytosol] + Diphosphate[cytosol]
Isopentenyl diphosphate[cytosol] => Diphosphate[cytosol]
trans,trans-Farnesyl diphosphate[cytosol] + Isopentenyl diphosphate[cytosol] => di-trans,poly-cis-Decaprenyl diphosphate[cytosol] + Diphosphate[cytosol]
Geranylgeranyl diphosphate[cytosol] + Isopentenyl diphosphate[cytosol] => di-trans,poly-cis-Decaprenyl diphosphate[cytosol] + Diphosphate[cytosol]
all-trans-Octaprenyl diphosphate[cytosol] + Isopentenyl diphosphate[cytosol] => di-trans,poly-cis-Decaprenyl diphosphate[cytosol] + Diphosphate[cytosol]
all-trans-Nonaprenyl diphosphate[cytosol] + Isopentenyl diphosphate[cytosol] => di-trans,poly-cis-Decaprenyl diphosphate[cytosol] + Diphosphate[cytosol]
all-trans-Heptaprenyl diphosphate[cytosol] + Isopentenyl diphosphate[cytosol] => trans,trans-Farnesyl diphosphate[cytosol] + Diphosphate[cytosol]
all-trans-Hexaprenyl diphosphate[cytosol] + Isopentenyl diphosphate[cytosol] => trans,trans-Farnesyl diphosphate[cytosol] + Diphosphate[cytosol]
all-trans-Pentaprenyl diphosphate[cytosol] + Isopentenyl diphosphate[cytosol] => trans,trans-Farnesyl diphosphate[cytosol] + Diphosphate[cytosol]
di-trans,poly-cis-Decaprenyl diphosphate[cytosol] + Isopentenyl diphosphate[cytosol] => trans,trans-Farnesyl diphosphate[cytosol] + Diphosphate[cytosol]
Isopentenyl diphosphate[cytosol] => Diphosphate[cytosol]
Geranylgeranyl diphosphate[cytosol] + Isopentenyl diphosphate[cytosol] => trans,trans-Farnesyl diphosphate[cytosol] + Diphosphate[cytosol]
all-trans-Octaprenyl diphosphate[cytosol] + Isopentenyl diphosphate[cytosol] => trans,trans-Farnesyl diphosphate[cytosol] + Diphosphate[cytosol]
all-trans-Heptaprenyl diphosphate[cytosol] + Isopentenyl diphosphate[cytosol] => Geranylgeranyl diphosphate[cytosol] + Diphosphate[cytosol]
all-trans-Hexaprenyl diphosphate[cytosol] + Isopentenyl diphosphate[cytosol] => Geranylgeranyl diphosphate[cytosol] + Diphosphate[cytosol]
all-trans-Pentaprenyl diphosphate[cytosol] + Isopentenyl diphosphate[cytosol] => Geranylgeranyl diphosphate[cytosol] + Diphosphate[cytosol]
di-trans,poly-cis-Decaprenyl diphosphate[cytosol] + Isopentenyl diphosphate[cytosol] => Geranylgeranyl diphosphate[cytosol] + Diphosphate[cytosol]
Isopentenyl diphosphate[cytosol] => Diphosphate[cytosol]
all-trans-Octaprenyl diphosphate[cytosol] + Isopentenyl diphosphate[cytosol] => Geranylgeranyl diphosphate[cytosol] + Diphosphate[cytosol]
all-trans-Nonaprenyl diphosphate[cytosol] + Isopentenyl diphosphate[cytosol] => Geranylgeranyl diphosphate[cytosol] + Diphosphate[cytosol]
all-trans-Heptaprenyl diphosphate[cytosol] + Isopentenyl diphosphate[cytosol] => all-trans-Octaprenyl diphosphate[cytosol] + Diphosphate[cytosol]
all-trans-Hexaprenyl diphosphate[cytosol] + Isopentenyl diphosphate[cytosol] => all-trans-

Octaprenyl diphosphate[cytosol] + Diphosphate[cytosol]
all-trans-Pentaprenyl diphosphate[cytosol] + Isopentenyl diphosphate[cytosol] => all-trans-Octaprenyl diphosphate[cytosol] + Diphosphate[cytosol]
di-trans,poly-cis-Decaprenyl diphosphate[cytosol] + Isopentenyl diphosphate[cytosol] => all-trans-Octaprenyl diphosphate[cytosol] + Diphosphate[cytosol]
trans,trans-Farnesyl diphosphate[cytosol] + Isopentenyl diphosphate[cytosol] => all-trans-Octaprenyl diphosphate[cytosol] + Diphosphate[cytosol]
Geranylgeranyl diphosphate[cytosol] + Isopentenyl diphosphate[cytosol] => all-trans-Octaprenyl diphosphate[cytosol] + Diphosphate[cytosol]
Isopentenyl diphosphate[cytosol] => Diphosphate[cytosol]
all-trans-Nonaprenyl diphosphate[cytosol] + Isopentenyl diphosphate[cytosol] => all-trans-Octaprenyl diphosphate[cytosol] + Diphosphate[cytosol]
all-trans-Heptaprenyl diphosphate[cytosol] + Isopentenyl diphosphate[cytosol] => all-trans-Nonaprenyl diphosphate[cytosol] + Diphosphate[cytosol]
all-trans-Hexaprenyl diphosphate[cytosol] + Isopentenyl diphosphate[cytosol] => all-trans-Nonaprenyl diphosphate[cytosol] + Diphosphate[cytosol]
all-trans-Pentaprenyl diphosphate[cytosol] + Isopentenyl diphosphate[cytosol] => all-trans-Nonaprenyl diphosphate[cytosol] + Diphosphate[cytosol]
di-trans,poly-cis-Decaprenyl diphosphate[cytosol] + Isopentenyl diphosphate[cytosol] => all-trans-Nonaprenyl diphosphate[cytosol] + Diphosphate[cytosol]
trans,trans-Farnesyl diphosphate[cytosol] + Isopentenyl diphosphate[cytosol] => all-trans-Nonaprenyl diphosphate[cytosol] + Diphosphate[cytosol]
Geranylgeranyl diphosphate[cytosol] + Isopentenyl diphosphate[cytosol] => all-trans-Nonaprenyl diphosphate[cytosol] + Diphosphate[cytosol]
all-trans-Octaprenyl diphosphate[cytosol] + Isopentenyl diphosphate[cytosol] => all-trans-Nonaprenyl diphosphate[cytosol] + Diphosphate[cytosol]
Isopentenyl diphosphate[cytosol] => Diphosphate[cytosol]
NADPH[cytosol] + Cholesterol[cytosol] + H+[cytosol] + Oxygen[cytosol] => NADP+[cytosol] + Cerebrosterol[cytosol] + H2O[cytosol]
Reduced FMN[cytosol] + NAD+[cytosol] => FMN[cytosol] + NADH[cytosol] + H+[cytosol]
NADPH[cytosol] + 6-Lactoyl-5,6,7,8-tetrahydropterin[cytosol] + H+[cytosol] => NADP+[cytosol] + Tetrahydrobiopterin[cytosol]
L-Threonine[cytosol] => 2-Oxobutanoate[cytosol] + H+[cytosol] + NH3[cytosol]
D-Ribulose 5-phosphate[cytosol] => D-Ribose 5-phosphate[cytosol]
leukotriene-D4[cytosol] + H2O[cytosol] => leukotriene-E4[cytosol] + Glycine[cytosol]
Amylose[cytosol] => Glycogen[cytosol]
