# Supplementary material for: Reconstruction of Genome-Scale Active Metabolic Networks for 69 Human Cell Types and 16 Cancer Types Using INIT
Source: PLoS Comput Biol. 2012 May 17;8(5):e1002518. doi: 10.1371/journal.pcbi.1002518 (PMC3355067; doi:10.1371/journal.pcbi.1002518)
Supplement: Table S6 — List of genes for which their corresponding reactions were significantly more present in cancer tissues compared to their corresponding normal tissues (p-value<10e-4). (PDF) [file pcbi.1002518.s008.pdf]

Table S6. List of genes for which their corresponding reactions were significantly more present in cancer tissues compared to their corresponding normal tissues (p-value < 10e-4).

Ensembl Gene ID
ENSG00000177156
ENSG00000137825
ENSG00000102780
ENSG00000111339
ENSG00000136143
ENSG00000069943
ENSG00000161031
ENSG00000135423
ENSG00000167261
ENSG00000167261
ENSG00000175505
ENSG00000184752
ENSG00000090013
ENSG00000124767
ENSG00000114480
ENSG00000114480
ENSG00000111885
ENSG00000158008
ENSG00000036530
ENSG00000153574
