# Supplementary material for: Reconstruction of Genome-Scale Active Metabolic Networks for 69 Human Cell Types and 16 Cancer Types Using INIT
Source: PLoS Comput Biol. 2012 May 17;8(5):e1002518. doi: 10.1371/journal.pcbi.1002518 (PMC3355067; doi:10.1371/journal.pcbi.1002518)
Supplement: Table S7 — List of Reporter Metabolites (p-value<10e-4). (PDF) [file pcbi.1002518.s009.pdf]

Table S7. List of Reporter Metabolites (p-value < 10e-4)

Metabolite Name[compartment]
Isopentenyl diphosphate[cytosol]
di-trans,poly-cis-Decaprenyl diphosphate[cytosol]
all-trans-Hexaprenyl diphosphate[cytosol]
all-trans-Heptaprenyl diphosphate[cytosol]
all-trans-Octaprenyl diphosphate[cytosol]
all-trans-Nonaprenyl diphosphate[cytosol]
all-trans-Pentaprenyl diphosphate[cytosol]
Geranylgeranyl diphosphate[cytosol]
H2O2[extracellular]
spermidine monoaldehyde 2[extracellular]
spermidine monoaldehyde 1[extracellular]
Phenethylamine[peroxisomes]
spermine monoaldehyde[extracellular]
Reduced riboflavin[cytosol]
Cerebrosterol[cytosol]
Oxygen[extracellular]
spermidine dialdehyde[extracellular]
1-Methylpyrrolinium[extracellular]
5-Aminopentanal[extracellular]
Cadaverine[extracellular]
N-Methylputrescine[extracellular]
NH4+[extracellular]
NH3[peroxisomes]
2-Acyl-sn-glycero-3-phosphoethanolamine[extracellular]
glycerophosphoserine[extracellular]
Tyramine[peroxisomes]
Diphosphate[cytosol]
Spermidine[extracellular]
trans,trans-Farnesyl diphosphate[cytosol]
5-oxo-12(S)-hydroxy-eicosa-2E,8E,10E,14Z-tetraenoyl-CoA[peroxisomes]
Imidazole-4-acetaldehyde[extracellular]
timnodonate[peroxisomes]
Reduced FMN[cytosol]
sn-glycero-3-Phosphoethanolamine[extracellular]
sn-glycero-3-Phosphocholine[extracellular]
N-Acetyl-D-muramoate[extracellular]
N-Acetylmuramoyl-Ala[extracellular]
NH3[extracellular]
spermine dialdehyde[extracellular]
Bilirubin[extracellular]
1-Acyl-sn-glycero-3-phosphoethanolamine[lysosomes]

6-Lactoyl-5,6,7,8-tetrahydropterin[cytosol]
leukotriene-E4[cytosol]
beta-D-Glucuronosyl-(1->4)-N-acetyl-alpha-D-glucosaminylproteoglycan[cytosol]
N-Acetyl-alpha-D-glucosaminyl-(1->4)-beta-D-glucuronosyl-(1->4)-N-acetyl-alpha-D-glucosaminylproteoglycan[cytosol]
2-Amino-4-hydroxy-6-(erythro-1,2,3-trihydroxypropyl)dihydropteridine triphosphate[mitochondria]
Bilirubin[cytosol]
Biliverdin[cytosol]
(2S)-2-Methylacyl-CoA[peroxisomes]
(2R)-2-Methylacyl-CoA[peroxisomes]
Aminoacetone[cytosol]
Aminoacetone[peroxisomes]
L-2-Amino-3-oxobutanoic acid[cytosol]
quinonoid dihydrobiopterin[nucleus]
O2'-4a-cyclic-tetrahydrobiopterin[nucleus]
tetrahydrobiopterin-4a-carbinolamine[nucleus]
Chondroitin 4-sulfate[cytosol]
Dimethylallyl diphosphate[peroxisomes]
4-Hydroxyphenylacetaldehyde[peroxisomes]
7-Dehydrodesmosterol[cytosol]
D-myo-Inositol 3,4-bisphosphate[cytosol]
Fatty acid[extracellular]
Alprostadil[nucleus]
1,3-Diaminopropane[extracellular]
3-Aminopropanal[extracellular]
Phenylacetaldehyde[peroxisomes]
1-Acyl-sn-glycero-3-phosphocholine[lysosomes]
1,4-benzothiazine-o-quinonimine[cytosol]
Triphosphate[mitochondria]
Spermine[extracellular]
Methylglyoxal[peroxisomes]
(5Z)-(15S)-11alpha-Hydroxy-9,15-dioxoprostanoate[cytosol]
prostaglandin H1[nucleus]
