# Supplementary material for: Reconstruction of Genome-Scale Active Metabolic Networks for 69 Human Cell Types and 16 Cancer Types Using INIT
Source: PLoS Comput Biol. 2012 May 17;8(5):e1002518. doi: 10.1371/journal.pcbi.1002518 (PMC3355067; doi:10.1371/journal.pcbi.1002518)
Supplement: Table S8 — Versions of the databases used in the creation of the Human Metabolic Reaction database (HMR). (PDF) [file pcbi.1002518.s010.pdf]

Table S8. Versions of the databases used in the creation of the Human Metabolic Reaction database (HMR).

Database	Export format	Version	Downloaded from
Recon 1	Flat file, SBML	Jan 31, 2008	http://bigg.ucsd.edu/
EHMN	Excel	June 6, 2009	
HumanCyc	Flat file	12.5	http://biocyc.org/download.shtml
KEGG	Flat file, KGML	48	ftp://ftp.genome.jp/pub/kegg/*
HPA	Flat file	7.1	http://www.proteinatlas.org/
HMDB	Flat file	2.4	http://www.hmdb.ca/
BioGPS	Flat file	2.0	http://www.biogps.org/
HepatoNet1	Flat file	March 1, 2011	http://www.nature.com/msb/journal/

*The KEGG FTP has moved to http://www.bioinformatics.jp and requires a subscription.
