# Supplementary material for: Reconstruction of Genome-Scale Active Metabolic Networks for 69 Human Cell Types and 16 Cancer Types Using INIT
Source: PLoS Comput Biol. 2012 May 17;8(5):e1002518. doi: 10.1371/journal.pcbi.1002518 (PMC3355067; doi:10.1371/journal.pcbi.1002518)
Supplement: Table S9 — Number of the genes after each pre-processing step during the generation of iHuman1512 . Since the INIT algorithm provides a connected and functional model, reactions that are unconnected in the template model can never be included. In order to separate between reactions that were excluded due to connectivity reasons and those that were excluded due to negative evidence, a number of preprocessing steps were performed on the data in the HMR database. In the first step reactions that contain very generic metabolites such as “lipid” or “alcohol” were removed. Reactions that were elementally unbalanced were fixed or removed. Simulations were performed to ensure that the network could not gain carbon, energy or redox power in an unbalanced manner. In the second step reactions where directionality information was lacking were removed. In the third step reactions where one or more of the substrates could not be synthesized through some other reaction (unconnected reactions) were removed. Finally, in the fourth step reactions that couldn't carry flux when the model had access to all exchange metabolites (as defined in the EHMN) were removed. Consequently, iHuman1512, a connected human network with 5,535 reactions, associated with 1512 protein coding genes, was generated. The number of genes associated to the remaining reactions after each pre-processing steps are presented below. (PDF) [file pcbi.1002518.s011.pdf]

Table S9. Number of the genes after each pre-processing step during the generation of *iHuman1512*. Since the INIT algorithm provides a connected and functional model, reactions that are unconnected in the template model can never be included. In order to separate between reactions that were excluded due to connectivity reasons and those that were excluded due to negative evidence, a number of preprocessing steps were performed on the data in the HMR database. In the first step reactions that contain very generic metabolites such as "lipid" or "alcohol" were removed. Reactions that were elementally unbalanced were fixed or removed. Simulations were performed to ensure that the network could not gain carbon, energy or redox power in an unbalanced manner. In the second step reactions where directionality information was lacking were removed. In the third step reactions where one or more of the substrates could not be synthesized through some other reaction (unconnected reactions) were removed. Finally, in the fourth step reactions that couldn't carry flux when the model had access to all exchange metabolites (as defined in the EHMN) were removed. Consequently, *iHuman1512*, a connected human network with 5,535 reactions, associated with 1512 protein coding genes, was generated. The number of genes associated to the remaining reactions after each pre-processing steps are presented below.

Steps	Process	Genes
	Human Reaction Database **(HMR)**	2366
Step 1	After manually removing unbalanced reactions	2188
Step 2	After removing reactions with uncertain directionality	2129
Step 3	After removing unconnected reactions	1640
Step 4	After removing reactions which cannot carry flux **(*iHuman1512*)**	1512
