# Supplementary material for: Reconstruction of Genome-Scale Active Metabolic Networks for 69 Human Cell Types and 16 Cancer Types Using INIT
Source: PLoS Comput Biol. 2012 May 17;8(5):e1002518. doi: 10.1371/journal.pcbi.1002518 (PMC3355067; doi:10.1371/journal.pcbi.1002518)
Supplement: Table S10 — Comparison between iHuman1512 and some other published human metabolic networks. (PDF) [file pcbi.1002518.s012.pdf]

Table S10. Comparison between iHuman1512 and some other published human metabolic networks.

	Reactions	Metabolites	Genes
iHuman1512	5535	3397	1512
Recon 1	3402	2785	1496
EHMN	6216	2678	2480
HepatoNet1	2519	777	713
