# Supplementary material for: Reconstruction of Genome-Scale Active Metabolic Networks for 69 Human Cell Types and 16 Cancer Types Using INIT
Source: PLoS Comput Biol. 2012 May 17;8(5):e1002518. doi: 10.1371/journal.pcbi.1002518 (PMC3355067; doi:10.1371/journal.pcbi.1002518)
Supplement: Figure S1 — Clustering of 69 predicted cell type specific genome-scale metabolic models for normal tissues together with 16 for cancer tissues. A dendrogram generated by unsupervised hierarchical clustering of the models based on predicted gene presence and absence is shown. (PDF) [file pcbi.1002518.s001.pdf]

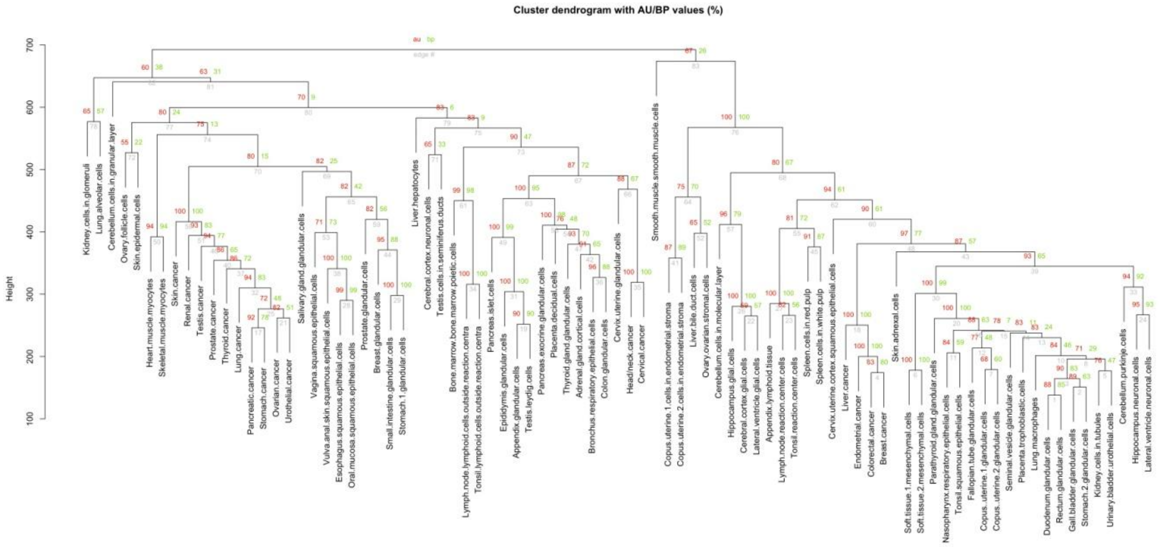

Figure S1. Clustering of 69 predicted cell type specific genome-scale metabolic models for normal tissues together with 16 for cancer tissues. A dendrogram generated by unsupervised hierarchical clustering of the models based on predicted gene presence and absence is shown.
